# Supplementary material for: Possible Role of Insulin-Degrading Enzyme in the Physiopathology of Retinitis Pigmentosa
Source: Cells. 2022 May 12;11(10):1621. doi: 10.3390/cells11101621 (PMC9139804; doi:10.3390/cells11101621)
Supplement: Supplementary file 1 [file cells-11-01621-s001.zip › cells-1649249-supplementary.pdf]

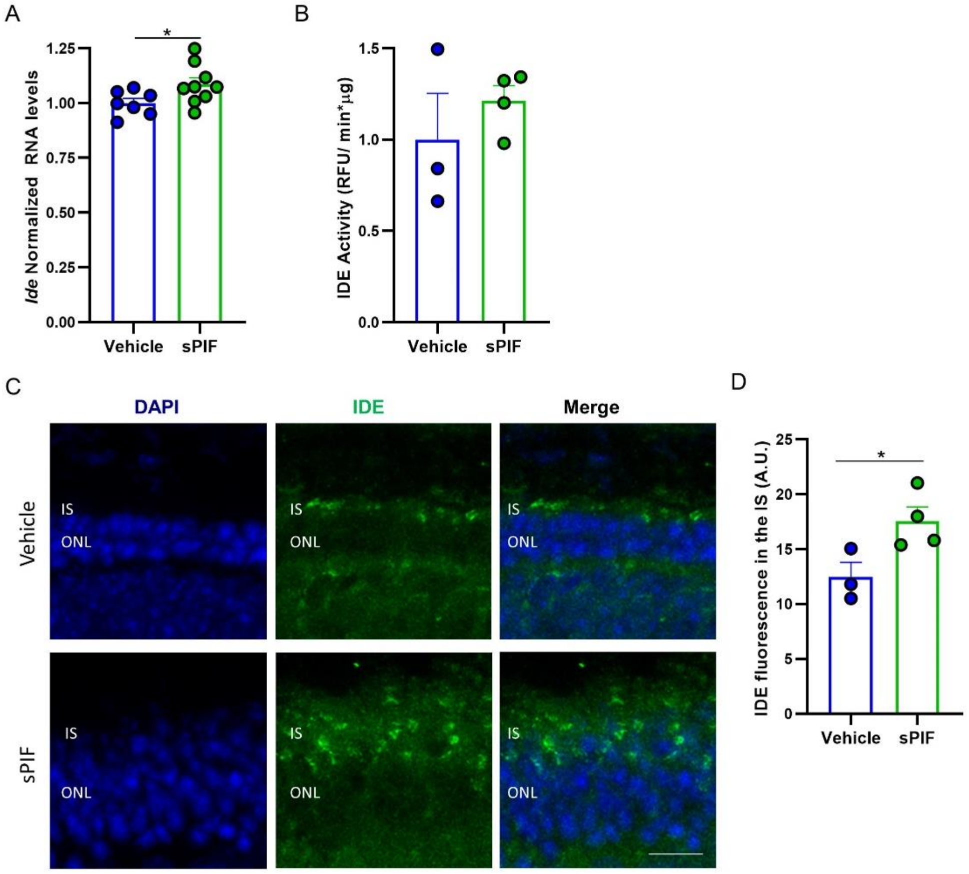

Supplementary Figure S1. Effect of sPIF on IDE levels. *rd10* mice received daily intraperitoneal injections of vehicle or sPIF. (**A)** RT-qPCR analysis of individual retinas from *rd10* animals treated from P15 to P25–28. **(B)** IDE activity measured by fluorometric assay in retinas of mice treated from P15–P28. Results represent the mean + SEM. *n*=7–9 individual retinas from 7–9 mice (A) or *n*= the mean of both retinas of 3–4 mice (B). (**C**) Representive images showing retinal sections at P29 immunostained for IDE (green) from *rd10* animals treated from P15 to P25–28. Nuclei were stained with DAPI (blue). IS, inner segment; ONL, outer nuclear layer; AU, arbitrary units. Scale bar: 10 µm. **(D)** Quantification of IDE fluorescence in the cone IS (area under the curve, see Methods section) of the mice mentioned in C. n=3-4 individual retinas from 3-4 mice. *p<0.05 (unpaired 2-tailed Student's t test).

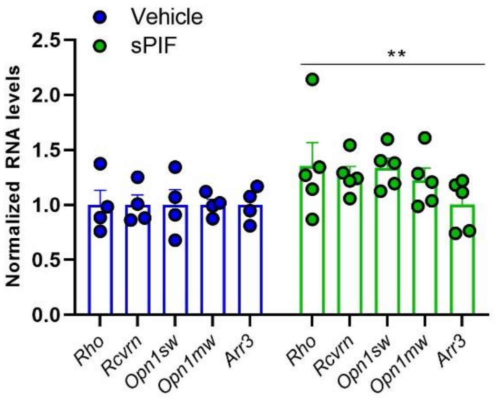

Supplementary Figure S2. Effect of sPIF treatment on photoreceptor gene expression. *rd10* mice received daily intraperitoneal injections of vehicle or sPIF from P15 to P27 inclusive. RT-qPCR analysis of individual retinas from sPIF- and vehicle-treated *rd10* mice at P29, after performing the ERG. Transcript levels were normalized to *Tbp* RNA levels and expressed relative to corresponding WT levels (= 1). Results represent the mean + SEM. *n*= 4-5 individual retinas from 4-5 mice. ***p*< 0.01 (2-way ANOVA). *Arr3,* cone arrestin; *Opn1mw,* L/M-Opsin; *Opn1sw,* S-Opsin; *Rcvrn,* Recoverin; *Rho,* Rhodopsin.
